# Preclinical studies of 5-fluoro-2′-deoxycytidine and tetrahydrouridine in pediatric brain tumors

**DOI:** 10.1007/s11060-015-1965-0

**Published:** 2015-10-30

**Authors:** Marie Morfouace, Birgit Nimmervoll, Nidal Boulos, Yogesh T. Patel, Anang Shelat, Burgess B. Freeman, Giles W. Robinson, Karen Wright, Amar Gajjar, Clinton F. Stewart, Richard J. Gilbertson, Martine F. Roussel

**Affiliations:** Department of Tumor Cell Biology, St. Jude Children’s Research Hospital, 262 Danny Thomas Place, Memphis, TN 38105 USA; CR UK Cambridge Institute, Li Ka Shing Centre, University of Cambridge, Robinson Way, Cambridge, CB2 ORE UK; Department of Pharmaceutical Sciences, St. Jude Children’s Research Hospital, 262 Danny Thomas Place, Memphis, TN 38105 USA; Department of Chemical Biology and Therapeutics, St. Jude Children’s Research Hospital, 262 Danny Thomas Place, Memphis, TN 38105 USA; Preclinical Pharmacokinetic Shared Resource, St. Jude Children’s Research Hospital, 262 Danny Thomas Place, Memphis, TN 38105 USA; Department of Oncology, St. Jude Children’s Research Hospital, 262 Danny Thomas Place, Memphis, TN 38105 USA

**Keywords:** G3 medulloblastoma, Ependymoma, Choroid plexus carcinoma, FdCyd, THU

## Abstract

Chemotherapies active in preclinical studies frequently fail in the clinic due to lack of efficacy, which limits progress for rare cancers since only small numbers of patients are available for clinical trials. Thus, a preclinical drug development pipeline was developed to prioritize potentially active regimens for pediatric brain tumors spanning from in vitro drug screening, through intracranial and intra-tumoral pharmacokinetics to in vivo efficacy studies. Here, as an example of the pipeline, data are presented for the combination of 5-fluoro-2′-deoxycytidine and tetrahydrouridine in three pediatric brain tumor models. The in vitro activity of nine novel therapies was tested against tumor spheres derived from faithful mouse models of Group 3 medulloblastoma, ependymoma, and choroid plexus carcinoma. Agents with the greatest in vitro potency were then subjected to a comprehensive series of in vivo pharmacokinetic (PK) and pharmacodynamic (PD) studies culminating in preclinical efficacy trials in mice harboring brain tumors. The nucleoside analog 5-fluoro-2′-deoxycytidine (FdCyd) markedly reduced the proliferation in vitro of all three brain tumor cell types at nanomolar concentrations. Detailed intracranial PK studies confirmed that systemically administered FdCyd exceeded concentrations in brain tumors necessary to inhibit tumor cell proliferation, but no tumor displayed a significant in vivo therapeutic response. Despite promising in vitro activity and in vivo PK properties, FdCyd is unlikely to be an effective treatment of pediatric brain tumors, and therefore was deprioritized for the clinic. Our comprehensive and integrated preclinical drug development pipeline should reduce the attrition of drugs in clinical trials.

**Electronic supplementary material:**

The online version of this article (doi:10.1007/s11060-015-1965-0) contains supplementary material, which is available to authorized users.

## Introduction

Brain tumors are the most common pediatric solid tumors, representing about 20 % of all childhood cancers. Treatment of brain tumors presents a major clinical challenge since the combination of neuro-surgery, radiation, and chemotherapy should be balanced with the risk of longterm neuroendocrine and neurocognitive side effects [1–4]. Medulloblastoma (MB), the most common malignant childhood brain tumor, includes four subtypes [WNT, SHH, Group 3 (G3), and G4], of which G3 has the worst prognosis [5–8]. Ependymoma (EPs) and choroid plexus carcinoma (CPCs) are less common and incurable in 40 and 70 % of cases, respectively [4, 9–11].

Conventional preclinical approaches to select drugs for clinical trial led to mixed results, with many drugs failing to reproduce in humans the anti-tumor activity observed in animal models. To better select and assess potential new therapies, we developed a series of mouse models that closely recapitulate the morphology, gene expression profile, and clinical behavior of MB [12–14], EP [9, 15], and CPC [16]. With these mouse models we performed high throughput drug screens (HTDS), in vivo pharmacokinetic (PK) and efficacy studies, to identify new therapies to treat children with brain tumors [2, 17, 18].

**Fig. 1:**
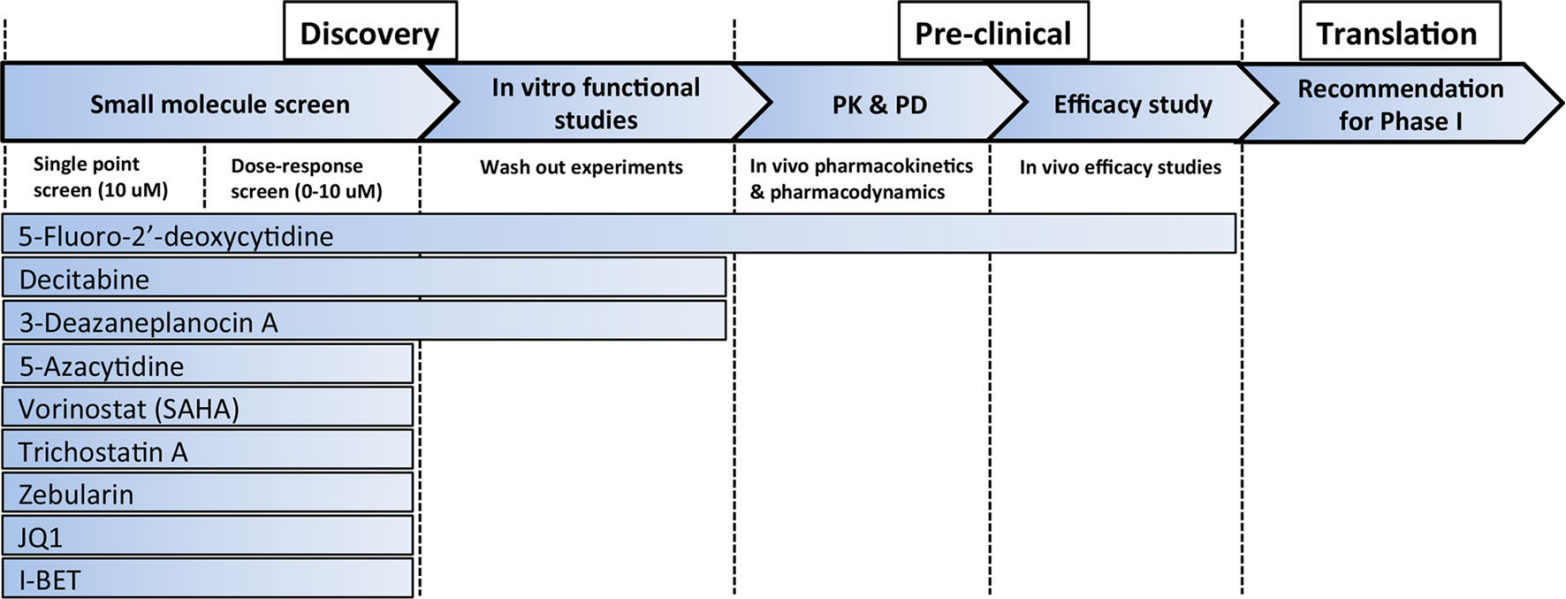
High-throughput screening, pharmacokinetics, and preclinical studies pipeline for pediatric brain tumors. Schematic drug pipeline to bring compounds from discovery (molecular screen, in vitro functional assays) to pre-clinical trials (PK and PD, efficacy studies) and translation in the clinic (recommendation for Phase I). Nine epigenetic compounds were tested in our pipeline. Only 5-fluoro-2′-deoxycytidine, our lead compound, performed successfully in all assays leading up to in vivo efficacy studies

Recent whole genome sequencing studies of pediatric brain tumors have identified few, recurrent oncogenic point mutations targetable therapeutically [8, 9, 12]. Rather, these tumors contain large chromosomal copy number changes or aberrant epigenomes. Therefore epigenetic regulators might provide attractive targets, since they may re-establish normal gene expression profiles, including those of tumor suppressors [19, 20]. We used a preclinical drug development pipeline (Fig. 1) to evaluate compounds that modulate the epigenome. Of the nine compounds tested, the nucleoside analogue 5-fluoro-2-deoxycytidine (FdCyd) showed the most activity in vitro. The results of extensive PK studies optimized the dosing and scheduling for preclinical efficacy studies. However, FdCyd co-administered with THU failed to produce significant tumor responses in vivo in each of the brain tumor models. Thus, the use of the combination of FdCyd and THU was deprioritized for the clinic providing an example of the utility of our drug development pipeline.

## Materials and methods

### Tissue culture

Neurospheres were derived from the cerebella of *Trp53*^*−*/*−*^, *Cdkn2c*^*−*/*−*^ mice (also referred as p53p18NS) [21] and from tumor cells from mouse G3 MBs (Myc 1 and Myc2) [13]. TB-12-5950 is a G3MB patient-derived xenograft (PDX) [18]. Ependymoma cells were generated and cultured as previously described [9]. 2889 is a PDX of ependymoma. CPC tumor cells were isolated from primary tumors in *Trp53*^*LoxP*^, *RB*^*LoxP*^, *Pten*^*LoxP*^ transgenic mice [16]. *Ink4a*/*Arf*^*−*/*−*^ neural stem cells were from the forebrain of E14.5 mouse embryos. The HEP G2, and BJ fibroblasts lines identified compounds with nonspecific toxicities [18]. (For more details see Supplementary Material).

### Library screen

In the Discovery Phase, the initial studies included the screen of a library of 9 compounds against epigenetic regulators at a single concentration and a dose-response on G3 MB, EP, and CPC tumor cells, as well as HEPG2 and BJ (Fig. 1) [17, 18]. The library and dose response studies are described in the Supplementary Material.

### In vitro functional studies

Myc1 and Myc2 G3 MB, EP, and CPC tumor cells (Supplementary Table 1) were plated in 96-well plates. After 24 h, 128 nL of a dilution series of a selected drug was transferred creating a final drug concentration of 0.5–9.3 µM. To assess the optimal exposure time, the drug was removed and replaced with fresh medium after 1, 3, 6, 10, 24, or 72 h (“wash-out”).

To test the effect of deoxycytidine on FdCyd efficacy, EP cells were plated in 96 well plates. After 24 h, FdCyd was added at final concentrations ranging from 0.1 nM to 50 μM alone or together with fixed concentration of deoxycytidine (Sigma) of 0.0122, 0.195, 6.25 or 100 μM.

In both experiments, 72 h after drug addition to cells, we added 100 µL of CellTiter-Glo reagent (Promega), and we read the luminescence signal. Data analysis used GraphPad Prism software (Version 5.04).

### In vivo pharmacokinetic studies

Once a drug passed the Discovery Phase it was moved to the Pre-Clinical Phase, which included PK and PD studies (Fig. 1). The details of the plasma PK and cerebral microdialysis studies are provided in the Supplemental Materials.

### In vivo efficacy studies

Efficacy studies (Fig. 1) were performed in G3 MB (Myc1), EP (915 RTBDN), and CPC (CPC300) cells co-expressing luciferase and yellow fluorescent protein (YFP) (vCL20SF2-Luc2aYFP). Drugs were injected post-tumor implant, after 4 days (G3 MB and EP), and 7 days (CPC). Tumor growth was monitored by bioluminescence imaging twice weekly. Complete blood counts, serum chemistries, and body weight were monitored in mice throughout therapy. Mice showing signs of morbidity, including head dome, slow motion, seizure, or toxicity (>20 % weight loss) were euthanized and tumors removed: one portion was fixed in 10 % formalin for histopathology and the other flash-frozen for molecular analysis. For more details, see Supplementary Material.

### In vitro and in vivo pharmacodynamic studies

To assess whether FdCyd/THU was cytotoxic or cytostatic, pharmacodynamic studies were performed on mouse G3 MB. G3 MB cells were plated and drugs added at their EC50. Cells were collected at 24, 48, and 72 h after drug addition and analyzed by fluorescence-activated cell sorting (FACS). Annexin V staining evaluated apoptosis, and DAPI staining DNA integrity. For proliferation analysis, cells were treated with FdCyd at the EC50 for 22 h and incubated with BrdU for an additional 2 h. Cells were analyzed for DNA content by FACS. To assess apoptosis and proliferation in vivo, G3 MB, EP, and CPC tumors were isolated from mice 3, 8, or 24 h after treatment with compound or vehicle, fixed in 10 % formalin, and sections were immunostained with antibodies to Caspase 3 (apoptosis) or Ki67 (proliferation). See Supplementary Material for more details.

### DNA methylation

DNA was extracted from mouse G3 MB neurospheres and tumors after DMSO or FdCyd and THU administration according to the manufacturer’s recommendation (Epigentek, P-1018). Global DNA methylation of 100 ng of DNA was measured by using a colorimetric kit that measures the level of 5-methylcytosine in an ELISA-like, microplate-based format (Epigentek, P-1034).

## Results

### FdCyd suppresses the in vitro proliferation of G3 MB, EP, and CPC neurosphere lines

**Fig. 2:**
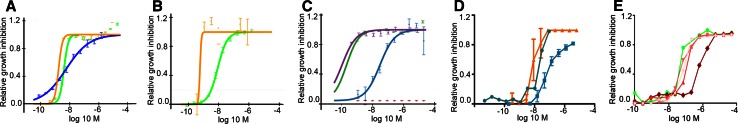
In vitro dose–response for FdCyd. Cells were plated at day 0. FdCyd was added in doses ranging from 1 to 10 µM at day 1; CellTiter-Glo assay results were read at day 3. **a** Mouse G3 MB Myc1 neurospheres EC_50_ = 1.7 nM (*orange curve*), mouse EP neurospheres EC_50_ = 4 nM (*green curve*), and mouse CPC neurospheres EC_50_ = 5.6 nM (*blue curve*); **b** Patient-derived xenografts of a human G3 medulloblastoma, TB-12-5950 EC_50_ = 1 nM (*orange curve*), and a human EP EC_50_ = 8.3 nM (*green curve*); **c** *Trp53* ^−/−^, *Cdkn2c* ^−/−^ neurospheres (*purple curve*), *Ink4Arf* ^−/−^ neural stem cells (*green curve*), BJ (*red curve*), and HEPG2 (*blue curve*) control cells. **d** 1 h FdCyd wash-out experiment in G3 MB (Myc1) (*orange curve*), EP (*green curve*), and CPC (*blue curve*) cells. **e** 72 h exposure in EP cells with increasing concentrations ranging from 0.1 to 50 μM of FdCyd alone (*green*) or with various fixed concentration of deoxycytidine: 100 μM (*dark red*); 6.25 μM (*red*); 0.195 μM (*pink*); 0.0122 μM (*blush pink*)

We tested the in vitro growth inhibition of nine epigenetic regulators against tumorspheres derived from murine G3 MB, EP, and CPC (Supplementary Table 2). We identified FdCyd as a highly effective inhibitor of proliferation of all three tumor cell types, with 72-hr EC_50_ values from 1 to 6 nM (Fig. 2a). FdCyd also efficiently suppressed the proliferation of tumor cells from PDXs of G3 MB and EP (Fig. 2b).

To assess anti-tumor selectivity, FdCyd growth inhibition assays were performed in several mouse and human cell lines (Fig. 2c). *Trp53*^−/−^, *Cdkn2c*^−/−^ neurospheres and *Ink4a*/*Arf*^−/−^ neural stem cells showed sensitivity to FdCyd, but not BJ and HEPG2 cells. EC_50_ values of additional mouse G3 MB and EP neurosphere lines displayed similar EC_50_ to those used in the primary screen (Supplementary Fig. 1A).

Because FdCyd had low EC50 values in mouse tumors with a good therapeutic index, it was chosen for in vitro functional studies that determined the concentration–time exposure of FdCyd required to suppress proliferation of G3 MB, EP, and CPC neurospheres. After 1 h FdCyd exposure, EC_50_ values were 8 ± 2 nM for G3 MB, 22 ± 8 nM for EP and 63 ± 28 nM for CPC (Fig. 2d). The remaining time points, showed that longer exposure to the drug decreased EC_50_ values as expected to low nanomolar: 5, 4, 2.3, and 1 nM (G3 MB, left panel), 17, 13, 3, and 0.7 nM (EP, middle panel) and 58, 44, 31, and 1 nM (CPC, right panel) (Supplementary Fig. 1B). To address whether the levels of deoxycytidine kinase (dCK) were sufficient to convert FdCyd to its active form, we added various concentrations of deoxycytidine, the endogenous substrate of dCK [22], to our cell culture media in combination with FdCyd and compared EC_50_ s to FdCyd alone (Fig. 2e). We found that 100 μM deoxycytidine in combination with FdCyd induced a shift in EC_50_ from 0.05 to 0.69 μM. In contrast, lower concentrations of deoxycytidine from 0.0122 up to 6.26 μM did not induce a dramatic shift in EC_50_ s.

FdCyd is a potent cytotoxic agent [23] and a DNA methyltransferase (DNMT) inhibitor in vitro [24]. In vivo, FdCyd is converted by cytidine deaminase into 5-fluoro-2-deoxyuridylate which impairs FdCyd-mediated DNMT inhibition [25]. To prevent the metabolism of FdCyd into its metabolites, a cytidine deaminase inhibitor, 3,4,5,6-tetrahydrouridine (THU) can be co-administered [25]. Full dose–response synergy experiments of THU and FdCyd against G3 MB neurospheres showed no antagonism between the two drugs, identifying FdCyd as the main active form in vitro (Supplementary Fig. 1C).

### Plasma pharmacokinetics and tumor extracellular fluid disposition of FdCyd

**Fig. 3:**
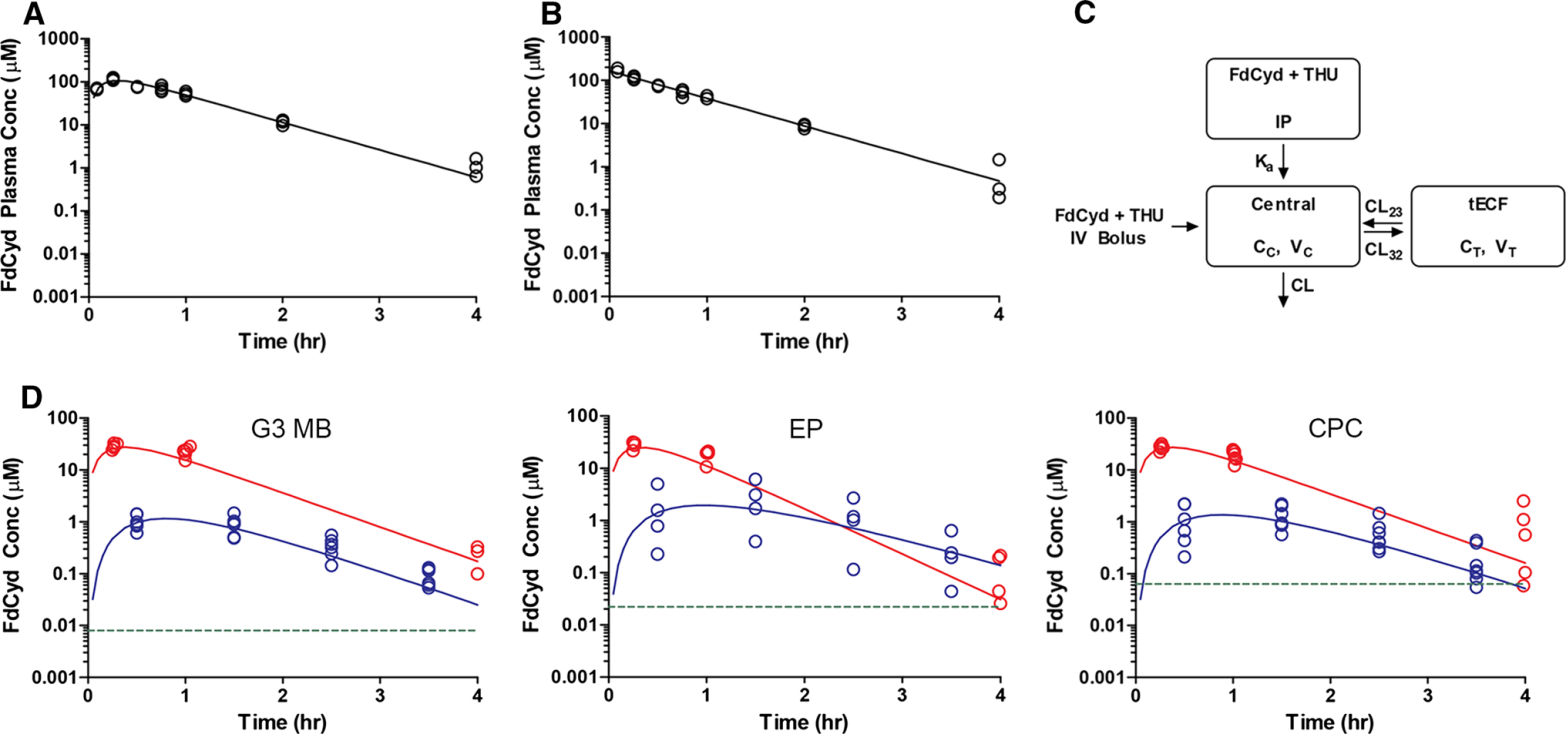
Plasma and tECF disposition of FdCyd. Full plasma pharmacokinetic study: unbound FdCyd concentrations in plasma are plotted against time for **a** IV and **b** IP administrations (*open circles* represents observed concentrations, *solid line* represents model-predicted population mean concentrations). **c** Schematic of pharmacokinetic model fitted to microdialysis and full plasma pharmacokinetic study results (K_a_: absorption rate constant after IP injection; CL: systemic clearance; CL_23_ and CL_32_: influx and efflux clearance for tECF compartment; V_C_ and V_T_: volume of central and tECF compartment). Cerebral microdialysis study: Unbound FdCyd concentrations in plasma and tECF plotted against time for CD1 nude mice bearing orthotopic **d** G3 MB, EP, or CPC tumors (*red open circle* and *solid line* represent observed and population mean concentrations of unbound FdCyd in plasma, respectively; *blue open circle* and *solid line* represent observed and population mean concentrations of unbound FdCyd in tECF, respectively; *green dotted line* represents in vitro 1 h IC_50_ in respective tumor model)

FdCyd plasma PK administered with THU was studied in non–tumor-bearing CD1 nude mice at a dosage of 25 mg/kg formulated with THU (100 mg/kg) [24] delivered either IV (Fig. 3a) or IP (Fig. 3b). FdCyd plasma concentration–time data were represented by a 1-compartment model (Fig. 3c). The limited plasma sampling time points derived by using a D-optimality method were 0.25, 1, and 4 h. The mean ± SD of individual plasma exposure (AUC_0-Inf_) of FdCyd in non–tumor-bearing CD1 nude mice after FdCyd (25 mg/kg) was 113.75 ± 4.77 µM*h after IV administration and 111.92 ± 6.55 µM*h after IP administration. Limited published clinical PK data suggested that FdCyd exposure in CD1 nude mice dosed at 25 mg/kg was approximately 4 times higher than that in humans given the recommended Phase II dosage (RP2D) of 100 mg/m^2^ [26]. Thus, assuming linear PK in mice, we reduced our IP mouse dosage to a more clinically relevant regimen of 6 mg/kg FdCyd in combination with 100 mg/kg THU. A confirmatory PK study (n  =  3, 3 time points per mouse), showed that the murine plasma exposure at this dosage was comparable to that estimated for the RP2D of FdCyd in humans. For subsequent microdialysis and efficacy studies, we used a FdCyd dosage of 6 mg/kg combined with 100 mg/kg THU.

We conducted individual cerebral microdialysis studies to assess FdCyd tECF disposition in CD1 nude mice bearing cortical implants of mouse G3 MB, EP, or CPC tumors (Fig. 3d). Population-based PK modeling derived individual plasma and tECF concentration–time profiles for each animal (Fig. 3c). FdCyd was negligibly bound to plasma protein (f_u,p_ ~ 1) and brain homogenate (f_u,b_ ~ 1) in vitro (data not shown). FdCyd tECF concentrations were above in vitro 1-h IC_50_ values for at least 3 h in all mouse tumor models, suggesting sufficient FdCyd exposure in the brain to inhibit tumor growth in vivo (Fig. 3d).

### FdCyd is ineffective in suppressing G3 MB, EP, and CPC tumor growth in vivo

**Fig. 4:**
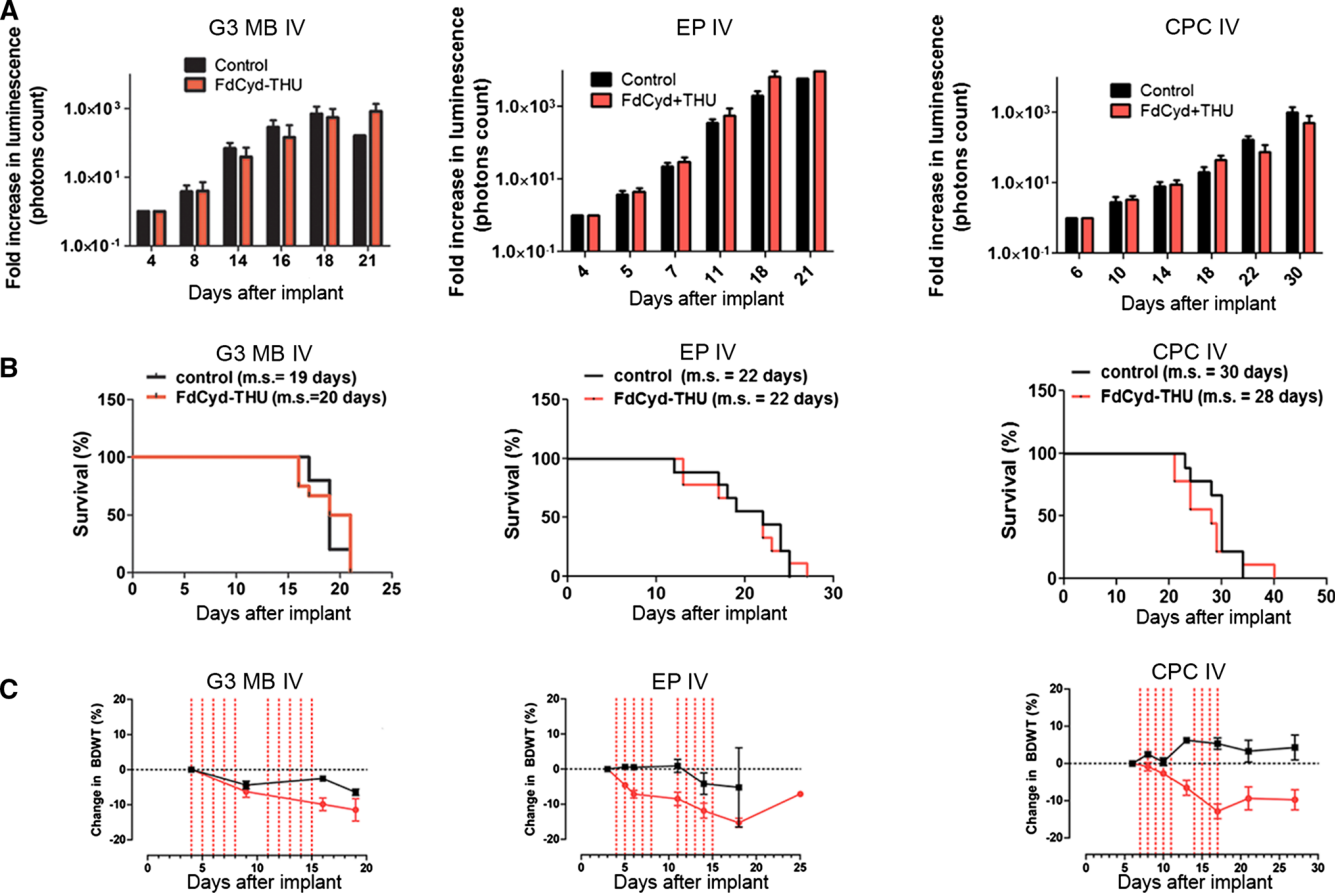
In vivo treatment of G3 MB-, EP-, and CPC-bearing mice with FdCyd and THU. **a** Fold-increase in bioluminescence signal of the brain of G3 MB-bearing mice (n  =  10 for FdCyd/THU-treated animals; n  =  5 for vehicle-treated animals), EP-bearing mice (n = 9 for FdCyd/THU-treated animals; n = 9 for vehicle-treated animals), and CPC-bearing mice (n = 9 for FdCyd/THU-treated animals; n = 9 for vehicle-treated animals). All animals were treated on days 5 through 9 and 12 through 16 after tumor implant with FdCyd (6 mg/kg) and THU (100 mg/kg) administered IV in a 200 µL volume of 5 % dextrose or with 200 μL of 5 % dextrose. **b** Survival curves for vehicle-treated animals (*black*) and FdCyd/THU-treated mice (*orange*): G3 MB-bearing mice, EP-bearing animals, or CPC-bearing animals all treated IV. **c** Body weight measurement in control (*black*) or treated animals (*orange*) for G3 MB, EP, and CPC

To determine if FdCyd was efficacious in vivo, mice were orthotopically implanted with luciferase-expressing mouse G3 MB, EP, or CPC tumorspheres. FdCyd dosing was modeled using our PK data that identified tECF concentrations above in vitro 1-h IC_50_ values in all tumor models when FdCyd was administered at 6 mg/kg together with 100 mg/kg THU. Dose scheduling was modeled on the clinical trial—NCT00978250, administered as a 5 days treatment followed by 2 days off for 2 weeks for a 4-week cycle. IV injections of FdCyd and THU did not decrease the luminescence signal of animals implanted with G3 MB (Fig. 4a, left panel), EP (Fig. 4a, middle panel) or CPC (Fig. 4a, right panel). Mice bearing mouse G3 MB had no increased survival when given IV FdCyd and THU compared to vehicle-treated animals, 19 versus 20 days, respectively (Fig. 4b). Mice bearing mouse EP or CPC (Fig. 4b) that received vehicle had a median survival of 22 and 30 days, respectively, but FdCyd and THU-treated mice had a median survival of 22 and 28 days, respectively. Some treated animals developed severe gut toxicity, manifested by diarrhea, and subsequent massive weight loss (11–18 %), requiring euthanasia before the end of the second treatment week in all 3 models (Fig. 4c). Complete blood counts including white blood cells, neutrophils, and platelets were performed on mouse G3 MB-bearing mice treated IV (Supplementary Fig. 2A) and on mouse EP-bearing mice treated IV (Supplementary Fig. 2B). No significant myelosuppression was observed.

### In vitro and in vivo FdCyd pharmacodynamic studies

**Fig. 5:**
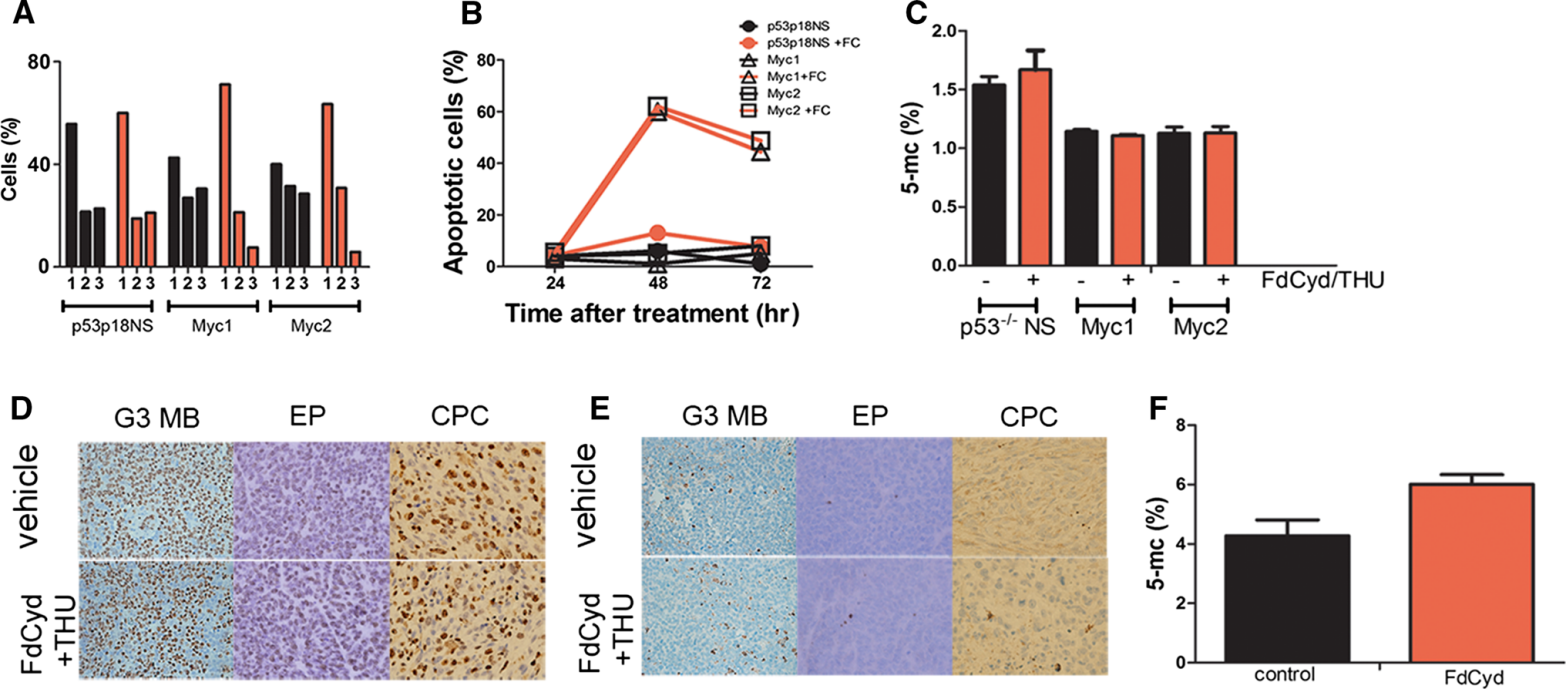
In vitro and in vivo pharmacodynamics studies**. a**, **b** In vitro: *Trp53* ^−/−^ *, Cdkn2c* ^−/−^ neurospheres and Myc1 and Myc2 G3 MB tumor spheres were untreated (*black lines*) or treated with 4 nM FdCyd (*orange lines*) and analyzed for **a** proliferation by BrdU analysis 24 h after treatment (*1* G0/G1 phase, *2* S phase, *3* M phase) and **b** apoptosis by Annexin V and DAPI staining 24, 48, and 72 h after treatment. **c** In vitro: *Trp53* ^−/−^ *, Cdkn2c* ^−/−^ neurospheres and Myc1, Myc2, Myc3 G3 MB tumor spheres were left untreated (*black bars*) or treated with 4 nM FdCyd and 10 µM THU for 72 h (*orange bars*) and analyzed for total DNA methylation (% 5-methyl-cytosine). **d** In vivo: sections of tumors from mice transplanted with G3 MB, EP, or CPC tumor spheres; stained with antibodies for Ki67 and Caspase 3 and harvested at 3, 8, or 24 h after vehicle or FdCyd and THU treatment. Representative image of **d** Ki67 and **e** Caspase-3 stains in all three tumor models at 3 h time point. Control depicted in *upper panel* and FdCyd + THU treated animals depicted in *lower panel*. **f** Global DNA methylation in 3 independently-derived mouse G3 MBs from mice left untreated (*black bars*) or treated with FdCyd and THU (*orange bars*)

To determine the mechanism by which FdCyd inhibited tumorsphere proliferation in vitro, we tested the effect of a 3-day drug exposure on Myc1 and Myc2, and *Trp53*^−/−^*, Cdkn2c*^−/−^ neurospheres. Treatment of mouse G3 MB tumorspheres with FdCyd alone increased the number of cells in the G0/G1phase of the cell cycle (Fig. 5a) and the number of apoptotic cells (Fig. 5b) compared to DMSO-treated cells without affecting control neurospheres. We could not detect changes in total DNA methylation after a 3-day in vitro treatment with FdCyd (4 nM) and THU (10 µM), measured by the percentage of 5-methyl-cytosine (Fig. 5c).

To assess why FdCyd proved ineffective in vivo, we used immunohistochemistry (IHC) to test if exposure to the drug impacted in vivo cell proliferation (Fig. 5d; Supplementary Table 3) or apoptosis (Fig. 5e; Supplementary Table 4), at 3, 8, or 24 h post-treatment with either vehicle or FdCyd and THU. Representative images for all three mouse tumor types showed Ki67 (Fig. 5d) and Caspase-3 (Fig. 5e) staining 3 h after treatment with vehicle or FdCyd and THU (all time points in Supplementary Fig. 3). Mouse G3 MB showed significant differences (p = 0.0022) between treated and untreated mice at 3 h, but this effect was lost over time. In vivo, we found no significant difference in the percentage of 5-methyl-cytosine in tumors from G3 MB-bearing animals vehicle-treated versus those treated with FdCyd and THU (Fig. 5f).

## Discussion

For a quarter of a century, no new drugs have been approved by the US Food and Drug Administration to treat children with brain tumors. The overall success rate in developing new agents from preclinical models to clinical cancer trials is less than 8 % [27]. To find new therapies, we used accurate mouse models of pediatric brain tumors that morphologically and transcriptionally recapitulate the human diseases. We devised a preclinical drug pipeline combining in vitro and in vivo screens focusing on epigenetic regulators. One of the key features of this pipeline was the early integration of toxicity in vitro through comparative studies with mouse neurospheres and embryonic neural stem cells, human HEP G2 and BJ cells. Despite activity against the neural stem cell population in vitro, no obvious neuro-toxicity was observed in vivo.

We found that the pyrimidine analog FdCyd suppressed proliferation of all three tumor models in vitro. To use the most clinically relevant dosing regimen in preclinical studies, we performed rigorous plasma PK studies to assess FdCyd exposure in murine plasma. Since the plasma FdCyd AUC at our initial dosage (25 mg/kg) exceeded that reported for the recommended Phase II human dosage [28], we reduced the FdCyd dosage to 6 mg/kg. Using dosages that are associated with clinically relevant plasma systemic exposures is critical because many preclinical studies show antitumor effects, but at supra-pharmacologic concentrations, and, by inference, systemic exposure that cannot be achieved safely in patients. In addition, one-quarter of molecules entering clinical trials fail due to pharmacological issues including the lack of absorption or penetration into the target organ [26, 27, 29]. We performed microdialysis studies to document adequate tECF exposure. Using a clinically relevant dosage and schedule, we saw no significant tumor inhibition in our tumor models. The FdCyd dosage and schedule was based upon the presumption that we would see activity in our models to rapidly move FdCyd into clinical trials for children with brain tumors. We were prevented from evaluating alternative schedules due to toxicities observed with our initial regimen.

FdCyd integrates into chromatin, inhibits DNA methylation and induces G2/M arrest in colon cancer cell lines [30]. In contrast, FdCyd induced G0/G1 arrest in vitro in the three mouse models, consistent with a report that suggests DNA-damaging agents can cause either G1- or G2-phase cell-cycle arrest [31]. We predicted that the FdCyd’s metabolite 5-FU [24] would lead to apoptosis, as shown in a previous ependymoma study [17]. We detected a significant increase in apoptosis in vitro after 48 h of treatment with FdCyd but only a slight, significant increase after 3 h in vivo in G3 MB. We found small, but not significant, increase of apoptosis in the CPC model, while at all other time points, we saw no difference between treated and untreated mice. None of the models treated with FdCyd and THU showed changes in proliferation. Since we did not see any cytotoxic effect in vivo compared to in vitro, the question remains whether the cytotoxic effect was insufficient to alter Caspase3 or Ki67 levels in vivo.

Much insight has been gained into the relevance and function of histone methylation-dependent epigenetic events in G3 MB [32] and EP [33], while little is known for CPC. Despite FdCyd’s ability to bind DNA methyltransferases and prevent DNA methylation [34], FdCyd and THU did not affect global DNA methylation in vitro or in vivo in mouse G3 MBs.

Ki67 and Caspase3 results highlight the difference between in vivo and in vitro cell behavior. Even though neurospheres, when implanted into the cortex of naive animals, recapitulate the primary tumors, the transcriptome and methylome of cells in culture might be different from those in vivo. Indeed, multiple studies have already addressed these differences [35]. Therefore, drug screening should be based on multiple cell lines and integrate a validation cohort of independently-derived tumors, as well as primary patient-derived xenografts, when available. Moving forward, it will be important to integrate pharmacodynamic measurements and potentially assess the mechanism of cell death in vitro as early as possible.

The lack of in vivo efficacy may be explained by chemoresistance that could be due to tumor cell-intrinsic changes [36], extrinsic factors such as cytokines and growth factors [37] emphasizing the importance of the tumor microenvironment, especially the presence of tumor-associated macrophages that could play a critical role in drug resistance [38]. Combination studies with epigenetic compounds may be a better therapeutic strategy than single-arm studies. Many new epigenetic drugs may offer synergistic benefits and synergize with conventional therapies [39].

Another valid hypothesis was that the lack of FdCyd efficacy in vivo might have been due to competition with deoxycytidine, the endogenous substrate of deoxycytidine kinase [22] since deoxycytidine is required to convert FdCyd into its active prodrug (FdCyd triphosphate) [24]. We found that only high levels (100 40]. Therefore, we conclude that it is unlikely that deoxycytidine concentrations reached levels high enough to affect FdCyd activation in our in vivo experiments.

Our studies highlight the importance of in vitro toxicity studies in combination with detailed PK and PD studies to identify drugs for use in the clinic and to avoid taking a drug forward that looks feasible in in vitro screens but for which efficacy does not translate into an in vivo setting. By testing multiple tumor model systems, including faithful mouse models and PDXs, we reduced the effects of bias and provided a more reliable readout. Using this approach, we recently identified an inhibitor of the ABC transporter ABCG2 to be efficacious in increasing survival of G3 MB-bearing mice [41], demonstrating that this pipeline allows the identification of novel therapies. Therefore, we propose to implement our preclinical screening pipeline as a standard of practice. In the future, candidate compounds will be tested in pre-clinical studies in tumor-bearing animals in a more clinically relevant setting by their integration with resection followed by radiation and standard-of-care chemotherapy.

## Electronic supplementary material

Supplementary material 1 (DOCX 18860 kb)
