# Supplementary material for: Preclinical studies of 5-fluoro-2′-deoxycytidine and tetrahydrouridine in pediatric brain tumors
Source: J Neurooncol. 2015 Oct 30;126:225–34. doi: 10.1007/s11060-015-1965-0 (PMC4718940; doi:10.1007/s11060-015-1965-0)
Supplement: Supplementary file 1 — Supplementary material 1 (DOCX 18860 kb) [file 11060_2015_1965_MOESM1_ESM.docx]

*Cancer Therapy: Preclinical*

**Morfouace et al.**

**Supplementary material data file**

**Supplementary Methods:** Description of the epigenetic compound library, neurosphere lines, dose response and synergy experiments, pharmacokinetics and pharmacodynamics and *in vivo* treatment regimen.

**Table 1:** Cell density of the three tumor types in 384- and 96-well plates

**Table 2:** Compounds targeting epigenetic regulators

**Table 3:** Quantification of Ki67 staining to measure *in vivo* proliferation in all three tumor models.

**Table 4:** Quantification of Caspase 3 staining to measure *in vivo* apoptosis in all three tumor models.

**Supplementary Figure 1:** Screening validation and FdCyd and THU synergy experiment.

**Supplementary Figure 2:** Blood chemistry of mice during *in vivo* IP and IV treatment with FdCyd and THU.

**Supplementary Figure 3:** Representative images for all three brain tumor types stained with Ki67 and Caspase 3.

**Supplementary references:**

**Supplementary Methods**

**Chemicals and drugs**

The epigenetic library studied contained: Decitabine (Sigma-Aldrich, St. Louis, MO, USA), 3-Deazaneplanocin (Santa Cruz, Dallas, TX, USA), 5-Azacytidine (Sigma-Aldrich St. Louis, MO, USA), Vorinostat (Toronto Research Chemicals, Toronto, Canada), Trichostatin A (AG Chemicals, San Diego, CA, USA) , Zebularine (EMD Millipore, Billerica, MA, USA), JQ1 (Selleck, Houston, TX, USA), I-BET (Selleck, Houston, TX, USA), FdCyd (TCI America, Portland, OR, USA). FdCyd and THU (Biovision, Milpitas, CA, USA) were generously provided by the National Cancer Institute.

**Human and mouse G3 MB, EP and CPC neurosphere lines**

*G3 MB tumor cell lines:* Myc1 and Myc2, two independently-derived mouse G3 MB tumor spheres, were generated from granule neural progenitors (GNPs) from the cerebella of postnatal day 7 *Trp53^-/-^, Cdkn2c^-/-^* mice infected with Myc and red fluorescent protein (RFP) expressing retroviruses subsequently orthotopically transplanted into the cortices of naïve recipient mice, as previously described [[1](#_ENREF_1)]. Tumor cells were isolated and grown as tumor spheres described previously [[2](#_ENREF_2)]. The G3 MB patient-derived xenograft (PDX) TB-12-5950, was generated from a primary patient tumor treated at SJCRH orthotopically transplanted into the cortices of immunocompromised mice [[2](#_ENREF_2)].

*EP tumor cell lines:* EP tumors were generated in mice from E14.5 embryonic neural stem cells (Blbp-eGFP, *Ink4a/Arf*^−/−^) infected with retroviruses encoding the RTBDN oncogene, the C11orfRELA fusion gene or the C11orfYAP1 fusion gene. The EP human PDX was generated from a patient with supratentorial ependymoma and implanted intracranially in immunocompromised mice. Tumors were harvested and digested with collagenase type IV and hyaluronidase for 30 min at 37°C to prepare single-cell suspensions. EP tumor cells were cultured in neurobasal medium (Invitrogen) containing 2 mM L-glutamine, N2 supplement (Invitrogen), B27 supplement (Invitrogen), 20 ng/mL human recombinant epidermal growth factor (hrEGF; Invitrogen), 20 ng/mL human recombinant basic fibroblast growth factor (hrbFGF; Invitrogen ) and 50 μg/mL BSA in 5% CO_2_.

*CPC tumor cell lines*: CPC tumor cells were isolated from primary tumors developed in *Trp53*^LoxP^, *RB*^LoxP^, *Pten* ^LoxP^ transgenic mice (Tong Y. and Gilbertson RJ., unpublished). Tumor tissue was digested with type IV collagenase and hyaluronidase for 30 min at 37°C, and single-cell suspensions prepared. Cells were isolated and grown as neurospheres in neurobasal medium containing 2 mM L-glutamine, 100 U/mL penicillin, 100 μg/mL streptomycin, N2 supplement (1%), B27 supplement (2%), 20 ng/mL EGF, 20 ng/mL bFGF and 50 μg/mL BSA in 5% CO_2_ (3). The mouse CPC tumor sphere line CPC300 derived from a primary mouse tumor was used for all *in vitro* and *in vivo* experiments.

**Dose response for candidate drug**

Briefly, cells (see Supplementary Table S2 for cell identity) were plated in 384-well plates (Corning) in 30 μL of supplemented neurobasal medium by using an automated plate filler (Wellmate, Matrix). After 24 hr, 28 nL of compound was transferred via pin tool, resulting in a final drug concentration of approximately 4 nM to 10 μM. After 72 hr of treatment, CellTiter-Glo reagent (Promega) was added, and the luminescence signal was measured using an automated Envision plate reader (Perkin-Elmer). Luminescence data were normalized by log10-transformation prior to calculating the percentage of inhibition using the following equation: 100 × (negative control mean − compound value)/(negative control mean − positive control mean). Our negative control represents cells treated with 0.1% DMSO with 0% inhibition. Cells treated with 35 μM cycloheximide (G3 MB tumor spheres) or 10 μM panobinostat (EP and CPC cells) were used as positive controls with 100% inhibition. The response of cells to the compounds were normalized to that of the positive controls present in each plate screened, shown in the Y axis of the dose-response curve. The EC_50_ indicates the drug concentration inducing 50% of the observed maximum inhibition reached by the compound (estimated by using Pipeline Pilot and R software). Curve fits were not attempted for compounds that did not achieve at least 50% inhibition relative to the positive control. Unless otherwise reported, the y-axis for each dose response curve in the figures was independently normalized to the range from 0 to 1 and therefore reported as ‘relative growth inhibition’. High through put assay data were analyzed by using our in-house Robust Interpretation of Screening Experiments (RISE) application that was written in Pipeline Pilot (Accelrys, v. 8.5) and R (R Development Core Team).

**Method for quantification of synergy data**

The method for quantification was performed as described in Stewart E et al. [[3](#_ENREF_3)]. Briefly, data in synergy experiment were fit using the following equation.


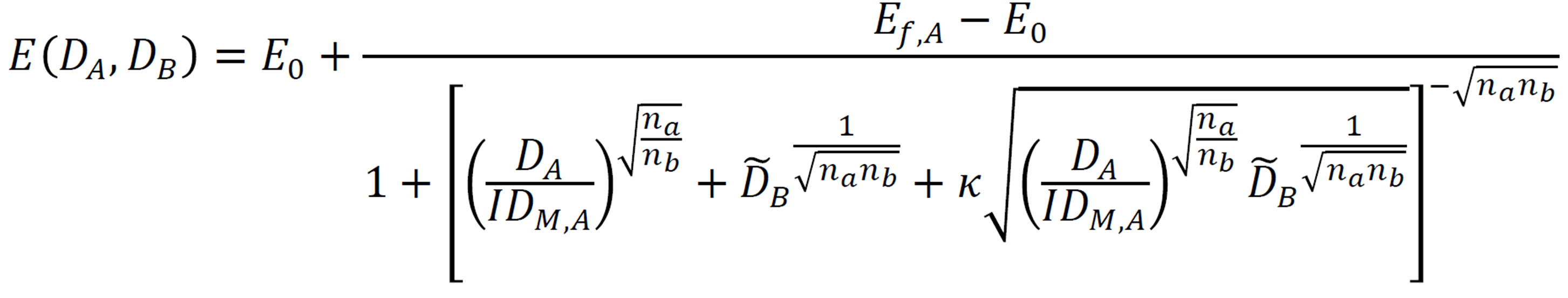


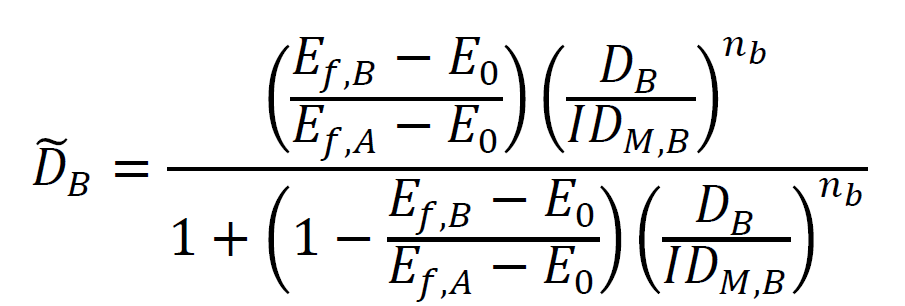


In this equation, D*_A_* and D*_B_* represent doses of drug A and drug B; *E*_0_ represents the predicted effect in the presence of no drugs; *E_f,A_* and *E_f,B_* represent the maximal effect of drug A and B. *ID_M,A_* and *ID_M,B_* represent the doses of half-maximal effect for drug A and B; *n_a_* and *n_b_* represent the Hill slopes of drug A and drug B, and κ is a parameter representing the level of interaction between drug A and drug B. Specifically, *κ* = 0 implies non-interaction, *κ* > 0 implies synergy, and *κ* < 0 implies antagonism.

**Bioanalytical method for quantifying FdCyd in plasma and Ringer’s solution**

The LC-MS/MS system for quantifying FdCyd consisted of an API 4000 mass spectrometer (AB SCIEX, Framingham, MA) equipped with a Prominence Ultra Fast Liquid Chromatograph (UFLC_XR_) system (Shimadzu, Canby, OR). FdCyd (purity 99.8%) was obtained from TCI America for use as an analytical standard. Gemcitabine-^13^C, ^15^N_2_ hydrochloride (98% purity, Toronto Research Chemicals) was used as internal standard (IS).

Chromatographic separation was achieved on a Phenomenex Synergi 4u fusion 80 A, 30 x 2.0 mm maintained at 40°C by using gradient elution. The mobile phases were 0.1% acetic acid in water and 0.1% acetic acid in acetonitrile. Mass spectrometric detection of FdCyd and IS was performed with the turbo ion spray in positive ionization with multiple reaction monitoring by using m/z transitions of 246.1>130 for FdCyd and 267.2>115 for IS. For plasma assays, the lower limit of quantification (LLOQ) was 2.5 ng/mL, with a calibration range of 2.5 ng/mL to 5000 ng/mL. For aCSF assays, the LLOQ was 1ng/mL, with a calibration range of 1ng/mL to 2000 ng/mL.

Mouse plasma samples were extracted by using acetonitrile (500 µL) to recover FdCyd and IS. The supernatants were evaporated to dryness, and the residues were reconstituted by using 900 µL of 10 mM ammonium acetate (pH 4.5). The artificial cerebrospinal fluid (aCSF) samples were mixed with 10 µL of IS (25 ng/mL), 25 µL of 15 mM ammonium bicarbonate (pH, 9.00; adjusted using ammonium hydroxide), and 100 μL of isopropanol. The resulting solutions were extracted by using ethyl acetate (700 µL). The supernatants (800 μL) were collected and evaporated to dryness. The dried extracts were reconstituted by using 150 µL of 10 mM ammonium acetate (pH 4.5) and then injected via the auto-sampler maintained at 15°C. The retention times of FdCyd and IS were found to be 2.06 ± 0.2 min and 2.03 ± 0.2 min, respectively. There were no endogenous interferences at the retention times of FdCyd and IS in plasma or aCSF.

The method was linear and reproducible, with typical *r* > 0.99 in both matrices. Precision and accuracies were assessed by running 6 replicates of high, medium, low, and LLOQ quality control samples in the relevant matrices (plasma assay, 4000, 750, 7.5, and 2.5 ng/mL, respectively; aCSF assay, 1600, 200, 3, and 1 ng/mL, respectively). The intra- and inter-day assay coefficients of variation (CV, %) were found to be ≤ 7.09%, and accuracies were found to be between 90.9% and 111%. Bench-top (6 hr at ambient temperature), short-term (48 hr at −20°C), and long-term (60 days at −20°C) temperature stabilities were assessed at low- and high-QC levels for both plasma and aCSF matrices. The mean accuracies of the low- and high-quality control samples ranged from 90.2% to 106%, and the %CVs were ≤ 5.35% for both matrices.

**Pharmacokinetic methodology**

An initial, detailed plasma PK study was performed in non–tumor-bearing CD1 nude mice to define drug disposition in our mouse model of interest, determine the plasma systemic exposure, and when possible, to compare the systemic exposure in the mouse with that achievable in patients at clinically relevant dosages. Dosing solutions were prepared in 5% dextrose for injection at concentrations providing a standard final dosing volume of 10 mL/kg (i.e., 2.5 mg/mL FdCyd and 10 mg/mL THU). Plasma samples were separated by centrifugation and stored at −80°C until analysis using a sensitive and specific liquid chromatography–tandem mass spectrometry (LC-MS/MS) method to determine FdCyd concentrations. Plasma samples were collected by using a sparse sampling design in which 2 - 3 blood samples per mouse were collected in tubes containing zebularine (final concentration in blood sample, 1 mg/mL) to inhibit cytidine deaminase activity *in vitro*.

An appropriate pharmacokinetic model was fit to the observed concentration-time data obtained from the initial PK studies (IP and IV administration) by using non-linear mixed-effect modeling (NONMEM 7.2, ICON Development Solutions). Using D-optimality implemented in ADAPT 5, we use the data from the plasma pharmacokinetic study to define a pharmacokinetic limited sampling model (LSM), which will be used in subsequent microdialysis studies [4,5]. Population mean parameters were estimated (standard error of estimation) for systemic clearance and volume of distribution at 0.90 (0.02) L/hr/kg for IV and 0.62 (0.03) L/kg for IP administration.

Cerebral microdialysis studies were performed in CD1 nude mice orthotopically transplanted with G3 MB, EP, or CPC tumors [,2,6]. Plasma and dialysate samples collected during microdialysis studies were analyzed by using LC-MS/MS to determine drug concentrations. Data obtained from microdialysis performed in individual tumor models were analyzed separately by using a compartmental approach. A model with a tECF compartment that was linked to the plasma compartment by using efflux (CL_32_) and influx (CL_23_) clearances was fit to the plasma and tumor ECF concentration-time data. These were obtained from previous plasma PK studies in all tumor models and from each microdialysis by using separate, tumor-specific population NONMEM 7.2.

The tECF-to-plasma partition coefficient of unbound drug (Kpt,uu) was calculated as a ratio of CL_23_ to CL_32_. The mean ± SD of individual tECF-to-plasma partition coefficients of unbound FdCyd (K_pt,uu_) in CD1 nude mice bearing G3 MB, EP, and CPC were 0.06 ± 0.02, 0.18 ± 0.14, and 0.09 ± 0.04, respectively. K_pt,uu_ values among different tumor types were not significantly different (ANOVA; *p* = 0.06). Inter-animal variability in K_pt,uu_, however, was higher in EP tumors than in G3 MB and CPC tumors.The area under plasma concentration-time curve (AUC_plasma_) was calculated as the ratio of dosage to CL, whereas AUC_tECF_ was calculated as the product of AUC_plasma_ and K_pt,uu_.

***In vivo* treatment of mouse bearing G3 MB, EP, and CPC**

Luciferase-expressing G3 MB tumor cells (1 × 10^5^) were resuspended in 5 µL of a 1:1 mix of Matrigel (BD Bioscience) and neurobasal medium; luciferase-expressing EP (1 × 10^4^) or CPC (2 × 10^3^) cells were resuspended in 5 µL Matrigel. Tumor cell suspensions were implanted in the cortices (for G3 MB and EP) or fourth ventricle (for CPC) of 6- to 8-week-old CD1 nude mice as previously described [1,2,6]. Tumor growth was assessed twice weekly by bioluminescence imaging. Briefly, mice were injected with 200 µL of a solution of D-Luciferin (15 mg/mL, Caliper Life Science) and imaged 5 min after injection by using an IVIS 200 *in vivo* imaging system (Xenogen, Caliper Life Science). An image showing light intensity (photons/second) was generated, and the signal in each mouse was quantified by using Living Image 4.0 software (Xenogen).

***In vitro* and *in vivo* pharmacodynamic studies**

G3 MB cells (1 × 10^3^) were plated in 24-well plates, and drugs were added at their EC50. Cells were collected at 24, 48, and 72 hr after drug addition to the culture medium and analyzed by fluorescence-activated cell sorting (FACS). Annexin V (550474, BD Pharmingen, CA) staining was used to evaluate apoptosis, and DAPI staining for DNA integrity. For proliferation analysis, cells were treated with FdCyd at the EC50 for 22 hr and incubated with BrdU (10 µM) for an additional 2 hr (552598, BD Pharmingen, CA). Cells were washed, prepared according to manufacturer’s recommendations (BD Pharmingen, 559619), and analyzed for DNA content by FACS. *In vivo* apoptosis and proliferation were performed on G3 MB, EP, and CPC tumors isolated from mice 3, 8, or 24 hr after treatment with compound or vehicle (saline). Tumors were fixed in 10% formalin. Sections (4-micron) were immunostained with antibodies to Caspase 3 (CP229C, BioCare Medical, Concorde, CA) to detect apoptotic cells, or Ki67 (ThermoShandon, RM9106) to detect proliferation. The number of positive cells for each stain was normalized to the total number of cells.

**Table 1. Cell density of the three tumor types in 384- and 96-well plates**

| **Tumor type** | **Cell line** | **384-well plate density (cells/well)** | **96-well plate density (cells/well)** |
| --- | --- | --- | --- |
| G3 MB | Myc1 m | 1000 | 2000 |
| G3 MB | Myc2 m | 1000 | 2000 |
| G3 MB | TB-12-5950 h | 3000 | 5000 |
| EP | 915 – RTBDN m | 750 | 1500 |
| EP | 5965 – RelAc11orf95 m | 3000 | 4000 |
| EP | 2889 h | 4000 | 5000 |
| WT | WT –E14.5 m | 2000 | 4000 |
| CPC | CPC300 m | 1000 | 1500 |

h, human ; m, mouse; WT, wild type; MB, medulloblastoma; EP, ependymoma; CPC, choroid plexus carcinoma

**Table 2. Compounds targeting epigenetic regulators.**

| **Identification No.** | **Compound** | **Mechanism of action** | **G3-MB EC_50_**  **[μM]** | **EP EC_50_**  **[μM]** | **CPC300 EC_50_ [μM]** |
| --- | --- | --- | --- | --- | --- |
| **SJ000785889** | **5-fluoro-2′-deoxycytidine** | DNMT inhibitor | 0.0017 | 0.004 | 0.0056 |
| **SJ000312371** | **Decitabine** | DNMT inhibitor | 0.96 | 0.5 | 6.7 |
| **SJ000785904** | **3-Deazaneplanocin A** | S-adenosylhomocysteine Hydrolase inhibitor, affect global methylation, including EZH2 and LSD1 activity | 0.36 | 0.11 | 0.31 |
| **SJ000285227** | **5-Azacytidine** | DNMT inhibitor | Inactive | 5.3 | 1.08 |
| **SJ000518939** | **SAHA (Vorinostat)** | HDAC inhibitor | Inactive | 6.3 | 6.7 |
| **SJ000142379** | **Trichostatin A** | HDAC inhibitor | 0.1 | 0.4 | 2.1 |
| **SJ000782349** | **Zebularin** | HDAC inhibitor | Inactive | Inactive | Inactive |
| **SJ000574790** | **JQ1** | BET inhibitor | 0.7 | 1.7 | >5.6 |
| **SJ000574791** | **I-BET** | BET inhibitor | 0.9 | 4.1 | >5.5 |

**Table 3. *In vivo* proliferation**

| **Ki67** |  | **V 3hr** | **FdCyd 3hr** | **V 8hr** | **FdCyd 8hr** | **V 24hr** | **FdCyd 24hr** |
| --- | --- | --- | --- | --- | --- | --- | --- |
| G3 MB | % of positive cells | 87.77 | 84.97 | 78.11 | 76.50 | 85.15 | 77.15 |
|  | SD | 16.13 | 8.996 | 9.488 | 8.839 | 8.305 | 11.13 |
| EP | % of positive cells | 81.79 | 77.06 | 80.10 | 72.88 | 68.41 | 72.62 |
|  | SD | 7.389 | 4.253 | 13.07 | 8.202 | 7.265 | 11.95 |
| CPC | % of positive cells | 59.03 | 47.82 | 62.25 | 23.4 | 53.18 | 61.3 |
|  | SD | 8.947 | 11.38 | 26.84 | 5.115 | 2.015 | 17.89 |

Mice bearing G3 MB, EP, or CPC tumors were harvested 3, 8, or 24 hr after IP injection of vehicle (V) or FdCyd + THU (FdCyd). Fixed brains were sliced and stained for Ki67. The percentage of positive cells was calculated based on total number of cells.

SD= standard deviation.

**Table 4. *In vivo* apoptosis**

| **Caspase-3** |  | **V 3hr** | **FdCyd 3hr** | **V 8hr** | **FdCyd 8hr** | **V 24hr** | **FdCyd 24hr** |
| --- | --- | --- | --- | --- | --- | --- | --- |
| G3 MB | % of positive cells | 12.37 | 19.88 | 8.945 | 11.04 | 9.419 | 10.38 |
|  | SD | 2.571 | 2.589 | 2.875 | 3.693 | 2.248 | 0.9008 |
| EPY | % of positive cells | 0.9975 | 1.416 | 1.110 | 1.210 | 1.427 | 1.030 |
|  | SD | 0.7418 | 0.9379 | 1.013 | 0.8946 | 0.4862 | 0.6563 |
| CPC | % of positive cells | 3.963 | 10.63 | 4.917 | 2.297 | 3.493 | 6.797 |
|  | SD | 2.286 | 5.77 | 1.76 | 0.8622 | 4.652 | 3.753 |

Mice bearing G3 MB, EP, or CPC tumors were harvested 3, 8, or 24 hr after IP injection of vehicle (V) or FdCyd + THU (FdCyd). Fixed brains were sliced and stained for caspase 3. The percentage of positive cells was calculated based on total number of cells. SD= standard deviation.

**Supplementary Figure 1. Screening validation and FdCyd and THU synergy experiment.** (A) 72 hr drug exposure of different mouse tumors. (B) Full FdCyd wash-out experiment at 1 hr (blue curve), 3 hr (green curve), 6 hr (orange curve), 10 hr (red curve), 24 hr (grey curve) and 72 hr (black curve) were performed in G3 MB (Myc1) (left panel), EP (middle panel), and CPC (right panel) cells. (C) Synergy study of FdCyd + THU in mouse G3 MB cells.

**

**

**Supplementary Figure 2. Blood chemistry of mice during *in vivo* IP and IV treatment with FdCyd and THU.** Quantification of white blood cells (WBC, top panels), neutrophils (middle panels), and platelets (bottom panels) in tumor-bearing animals treated with vehicle (black lines) and FdCyd + THU (red lines). Mice bearing (A) Group3 medulloblastoma (G3MB) and (B) ependymomas (EP) treated by intravenous (IV) injections.


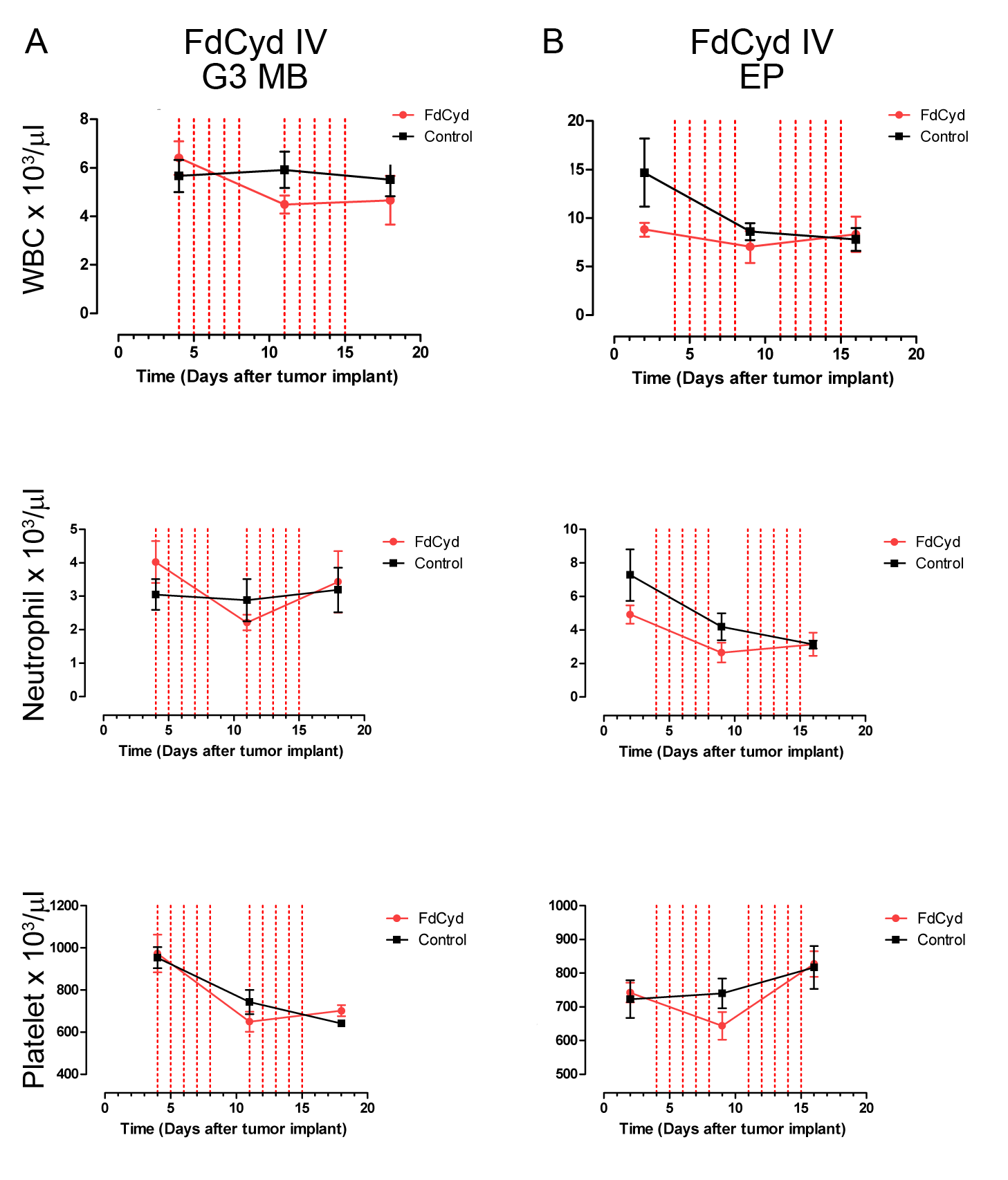


**Supplementary Figure 3. Representative images for all three brain tumor types stained with Ki67 and Caspase 3.** Immunohistochemistry of tumor sections with Ki67 to measure proliferation (A, C, E) and Caspase 3 to evaluate cell death (B, D, F) on tumors from animals treated with vehicle (top panels) or treated with FdCyd and THU (bottom panels) for 3, 8, or 24 hr. Representative images of tumor sections from G3 MB (A, B), EP (C, D), and CPC (E, F).

**
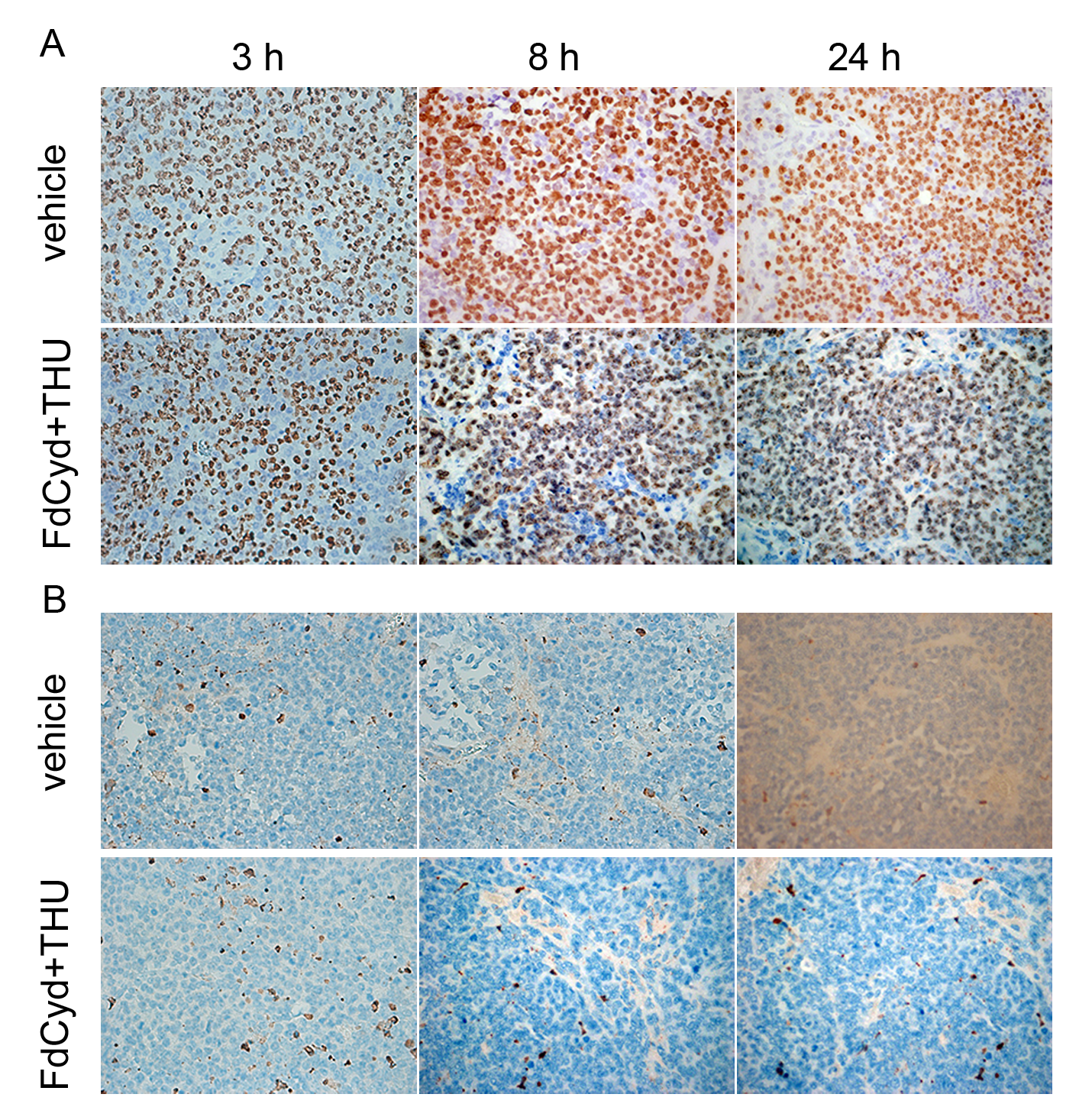

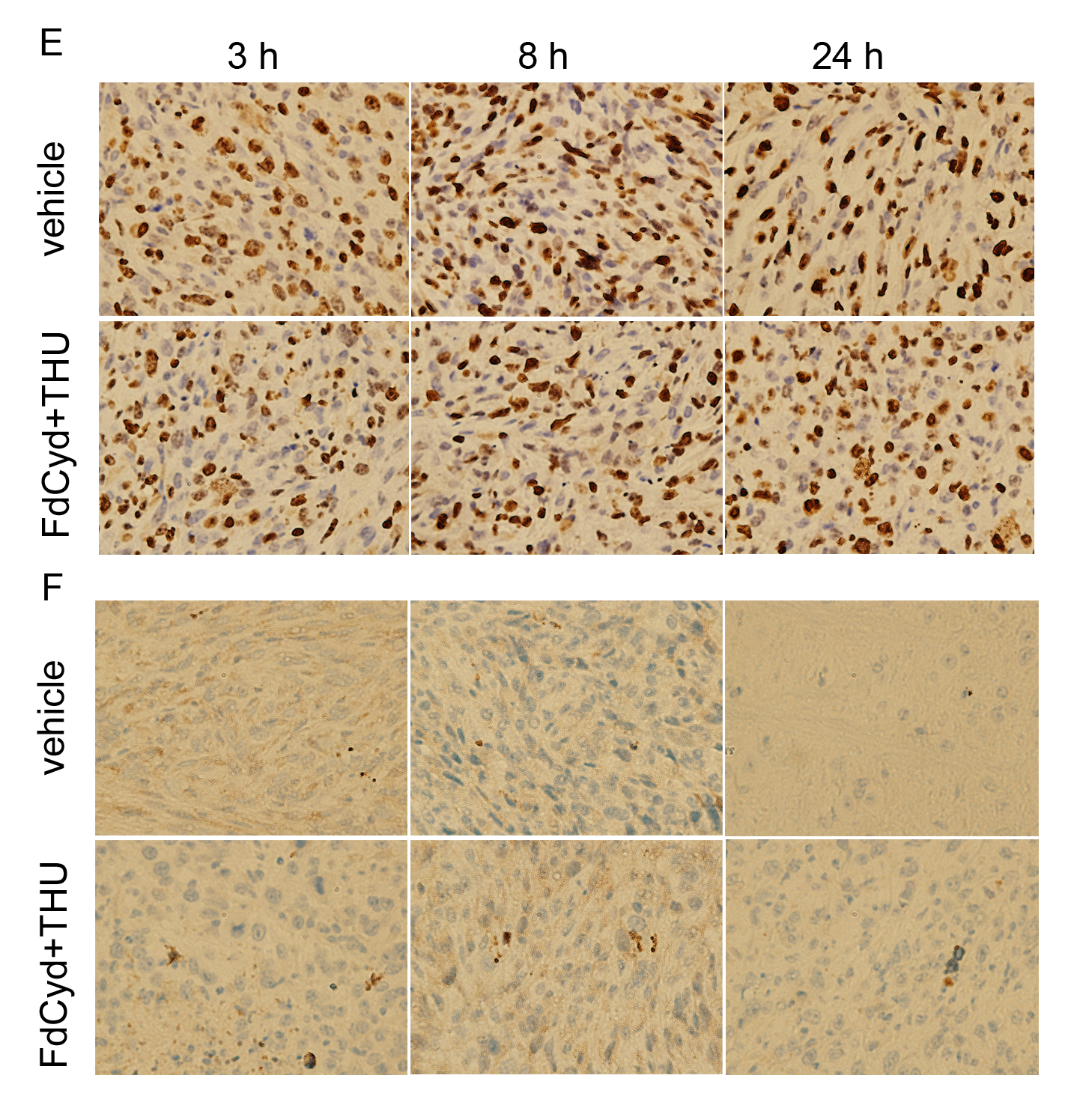
**

**
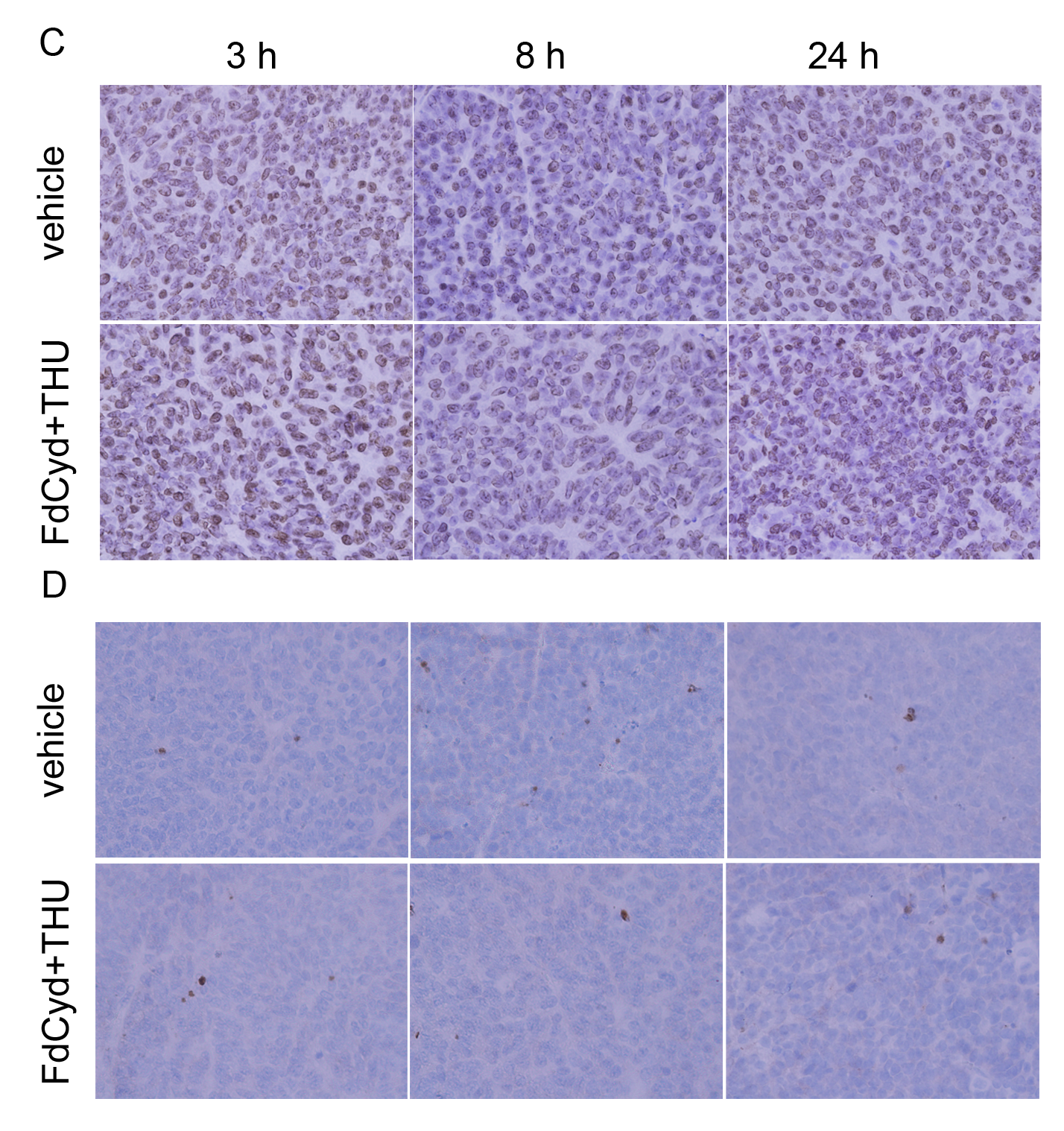
**

**Supplementary References**

1. Kawauchi D, Robinson G, Uziel T, Gibson P, Rehg J, Gao C, Finkelstein D, Qu C, Pounds S, Ellison DW, Gilbertson RJ, Roussel MF (2012) A mouse model of the most aggressive subgroup of human medulloblastoma. Cancer Cell 21 (2):168-180

2. Morfouace M, Shelat A, Jacus M, Freeman BB, III, Turner D, Robinson S, Zindy F, Wang YD, Finkelstein D, Ayrault O, Bihannic L, Puget S, Li XN, Olson JM, Robinson GW, Guy RK, Stewart CF, Gajjar A, Roussel MF (2014) Pemetrexed and gemcitabine as combination therapy for the treatment of Group3 medulloblastoma. Cancer Cell 25 (4):516-529

3. Stewart E, Goshorn R, Bradley C, Griffiths LM, Benavente C, Twarog NR, Miller GM, Caufield W, Freeman BB, III, Bahrami A, Pappo A, Wu J, Loh A, Karlstrom A, Calabrese C, Gordon B, Tsurkan L, Hatfield MJ, Potter PM, Snyder SE, Thiagarajan S, Shirinifard A, Sablauer A, Shelat AA, Dyer MA (2014) Targeting the DNA repair pathway in Ewing sarcoma. Cell Rep 9 (3):829-841

4. D'Argenio DZ, Schumitzky A, Wang X (2009) User's Guide: Pharmacokinetic/Pharmacodynamic Systems Analysis Software. Biomedical Simulations Resource. In.

5. Zhuang Y, Fraga CH, Hubbard KE, Hagedorn N, Panetta JC, Waters CM, Stewart CF (2006) Topotecan central nervous system penetration is altered by a tyrosine kinase inhibitor. Cancer Res 66 (23):11305-11313

6. Atkinson JM, Shelat AA, Carcaboso AM, Kranenburg TA, Arnold LA, Boulos N, Wright K, Johnson RA, Poppleton H, Mohankumar KM, Feau C, Phoenix T, Gibson P, Zhu L, Tong Y, Eden C, Ellison DW, Priebe W, Koul D, Yung WK, Gajjar A, Stewart CF, Guy RK, Gilbertson RJ (2011) An integrated in vitro and in vivo high-throughput screen identifies treatment leads for ependymoma. Cancer Cell 20 (3):384-399
